# Crisis management of COVID-19 at the primary healthcare level: challenges and solutions – a qualitative study

**DOI:** 10.1186/s12875-026-03379-2

**Published:** 2026-05-21

**Authors:** Mohammad-Hasan Imani-Nasab, Ebrahim Jaafaripooyan, Ali Mohammad Mosaddeghrad, Zahra Asadi-Piri

**Affiliations:** 1 Department of Public Health, School of Health and Nutrition, Social Determinants of Health Research Center, Lorestan University of Medical Sciences, Khorramabad, East Goldasht, Khorramabad, Iran; 2 Department of Health Management, Policy and Economics, School of Public Health, Tehran University of Medical Sciences, Enqelab Square, Tehran, Iran

**Keywords:** COVID-19, primary health care, Crisis management, Challenges

## Abstract

**Background:**

The COVID-19 outbreak, which began in December 2019, has become a global challenge, highlighting the need for effective crisis management solutions. This study aims to identify the challenges and crisis management Solutions related to COVID-19 at the primary healthcare level in Lorestan Province.

**Methods:**

This qualitative study adopted a phenomenological approach. Twenty-five healthcare managers (senior, middle, and operational) from the Public Health Deputy of Lorestan University of Medical Sciences and its affiliated health networks were selected through purposive and snowball sampling. Data were collected via semi-structured face-to-face interviews and analyzed using directed content analysis in MAXQDA software (version 10). A total of 375 initial codes were generated and organized into themes and subthemes. The trustworthiness of the findings was ensured using Guba and Lincoln’s criteria, including credibility (triangulation, member checking), dependability, confirmability, and transferability.

**Results:**

The main challenges identified at different stages of crisis management include: in the prevention stage, " awareness and understanding of the disease"; in the preparedness stage, " planning"; and in the response stage, "cultural-social, economic-political, governance challenges, and intersectoral cooperation. "Effective strategies and interventions proposed include: in the prevention stage, "strengthening the health and prevention sector"; in the preparedness stage, "managing human and informational resources, organization, and planning"; and in the response stage, " Strengthening human resource management, decision-making, and coordination."

**Conclusion:**

Primary healthcare faced major challenges in managing COVID-19, including poor coordination, unclear guidelines, and low public trust. To strengthen future responses, clear national protocols, transparent communication, improved inter-sectoral cooperation, and targeted support for vulnerable groups are essential. These measures can enhance preparedness and resilience in future health crises.

**Supplementary Information:**

The online version contains supplementary material available at 10.1186/s12875-026-03379-2.

## Background

Disasters and crises are sudden and unavoidable events that can severely disrupt a society’s ability to meet essential needs, deliver healthcare, and prevent financial and human losses—often necessitating international assistance [1]. The growing frequency and intensity of disasters lead to greater damage, higher mortality rates, and increased economic costs [2]. Economic losses from disasters have more than doubled over the past three decades, showing an increase of 145% from an average of around $70 billion per year in the 1990s to over $170 billion per year in the decade ending in 2020 [3]. The increasing frequency of disasters has led to growing concerns about healthcare preparedness, particularly for biological crises, which threaten public health and national security [4].

One of the most significant biological crises in recent history has been the Covid-19 pandemic. The outbreak was first reported in December 2019 in Wuhan, China, and due to its rapidly progressing pneumonia, it led to severe complications and high mortality rates [5]. On March 11, 2020, the World Health Organization (WHO) declared Covid-19 a global pandemic [6].With millions of cases and deaths worldwide, Covid-19 became one of the most devastating public health challenges in modern history [7]. As of September 2022, Iran had reported nearly 7.5 million cases and 144,000 deaths—the highest numbers in the Eastern Mediterranean region [8].

Within every country’s healthcare system, primary healthcare (PHC) plays a crucial role in crisis management, serving as the frontline in controlling disease outbreaks at the community level [9]. Effective management strategies in healthcare facilities before a crisis occurs can significantly enhance their response capacity [10]. Health centers must be well-prepared before disasters strike to ensure rapid and effective action when needed [11]. In fact, their level of preparedness can make a significant difference in reducing mortality rates and minimizing both physical and psychological harm [12].

According to a WHO report from March 26, 2020, PHC was identified as a cornerstone of the global response to Covid-19 [13]. In general healthcare policies, PHC serves as the foundation of healthcare systems, and during epidemics and pandemics, its role as the first line of defense becomes more critical than ever [14, 15]. This is especially true in low- and middle-income countries (LMICs), where access to hospitals and specialized care may be limited, making PHC an essential component of pandemic response efforts [16]. Globally, PHC is recognized as the backbone of an effective and equitable healthcare system, ensuring access to essential health services while addressing major public health challenges [17].

Research from Brazil indicates that in LMICs like Brazil, where there were insufficient prevention and control measures, the rapid spread of COVID-19 led to devastating outcomes [18].Therefore, during the COVID-19 crisis, PHC played an essential role in detecting and controlling the disease by distinguishing between respiratory illnesses and COVID-19 cases, enabling early diagnoses, offering support to vulnerable groups coping with health-related anxiety, and alleviating the burden on hospitals.

In Iran, Primary Health Care (PHC) serves as the cornerstone of the country’s health system and is delivered through a nationwide network of over 24,000 facilities. These services are structured within a multi-tiered referral system: at the first level, rural Health Houses and urban Health Posts provide basic preventive and primary care services; the second level comprises Comprehensive Health Service Centers (CHSCs), which offer outpatient care and supervise peripheral units; the third and fourth levels include specialized and teaching hospitals [19].

The COVID-19 pandemic—with its vast social, health, and economic implications—has created a unique opportunity to assess national and local crisis management capacities. Despite the central role of primary health care (PHC) in managing epidemics, multiple international studies have shown that PHC systems often face critical limitations during health crises. These include disruptions in essential services, poor integration with public health governance, inadequate crisis preparedness, and a lack of national-level coordination and support. Evidence from both high- and middle-income countries, such as Armenia and others across Europe, has highlighted how under-resourced and poorly integrated PHC infrastructures hindered timely and effective responses to COVID-19 [20–22]. In Iran, a qualitative study by Shami et al. (2025) explored PHC performance in managing COVID-19 and identified challenges across structural inputs, infectious disease control, policymaking, and community support [23].While that study provided valuable insight into PHC staff and expert perspectives, there remained a need for more localized and managerial-level analysis to inform targeted reforms.

However, there is a paucity of qualitative evidence exploring these challenges from the perspective of PHC managers, especially in low- and middle-income countries (LMICs), where structural limitations are often more severe. This qualitative study, based on a phenomenological approach, aims to explore the challenges and strategies of COVID-19 crisis management from the perspective of PHC managers in Lorestan Province, western Iran. By focusing on the experiences of managers at different levels (senior, mid-level, and operational), the study provides context-specific insights that can inform better planning, preparedness, and response in future health crises—especially in LMIC contexts. Connecting these local insights with international practices, this research adds to the growing body of evidence on PHC-based pandemic response.

## Methods

This study employed a qualitative phenomenological design, applying both inductive and deductive reasoning through a directed content analysis approach to explore the challenges and strategies of COVID-19 crisis management at the primary healthcare (PHC) level. The phenomenological method was chosen to gain a deep understanding of participants lived experiences, as this approach is well-suited for exploring the meanings and perspectives of individuals involved in complex phenomena. The study design was exploratory and tailored to answer open-ended research questions related to health crisis management.

The study setting included the Public Health Deputy of Lorestan University of Medical Sciences and four affiliated county health networks. The research population comprised senior, middle, and operational managers involved in disease prevention and management. Participants were selected using purposive and snowball sampling methods.

*Inclusion criteria* included: (1) at least one year of experience in COVID-19 management and (2) direct involvement in PHC decision-making.

*Exclusion criteria* included: (1) lack of willingness to participate or failure to provide informed consent (2), withdrawal during the interview process or incomplete participation (3), no direct involvement in COVID-19 response at the PHC level, and (4) inability to communicate effectively during interviews (e.g., due to health or language barriers). The initial estimate for data saturation was 30 interviews, but saturation was reached after 25 interviews and confirmed through ongoing discussions between two authors during the coding process.

### Data collection

Semi-structured interviews were conducted face-to-face and audio-recorded. Note-taking was also performed during interviews. An interview guide comprising approximately 30 open-ended questions was developed based on the four phases of crisis management: Prevention, Preparedness, Response, and Resilience. The questions covered a range of topics including infection control measures, facility preparedness, patient management during the COVID-19 crisis, workforce challenges, communication strategies, resource allocation, and lessons learned for future preparedness. A summary of the research proposal was provided to participants a few days in advance. Prior to interviews, the study objectives and relevance were explained. Interviews were conducted by a trained researcher and lasted approximately 60 min. For transparency and reproducibility, the full list of interview questions is provided in the supplementary materials (Appendix).

### Data analysis

We employed a directed content analysis approach to explore the challenges and strategies of COVID-19 crisis management at the primary healthcare (PHC) level. This approach integrated both deductive and inductive reasoning. The initial coding framework was guided by the four phases of crisis management—prevention, preparedness, response, and recovery. Simultaneously, we remained open to insights that emerged directly from the data, allowing for inductive coding to capture themes not encompassed within the predefined model.

All interviews were transcribed verbatim shortly after completion, and two researchers independently reviewed the transcripts to become deeply familiar with the content. They then coded the data separately using MAXQDA (version 10), applying the deductive framework while also identifying new codes and categories as they emerged. Coding differences were discussed collaboratively and resolved through consensus.

Through iterative coding and discussion, a total of 375 initial codes were identified and progressively grouped into subcategories and overarching themes. This process allowed for the integration of data-driven insights with theory-based structure. The analysis was thoroughly documented within the software environment, including analytic memos and coding decisions, to enhance transparency and auditability.

As the analysis progressed, comparisons were made across interviews to ensure thematic consistency and depth. Additional interviews were conducted when needed to enrich or clarify emerging patterns. This analytical process ensured that the data analysis was both systematic and flexible, grounded in participant experiences while aligned with established theoretical constructs. The combined use of deductive and inductive strategies supported the development of credible and contextually relevant findings.

### Trustworthiness and reliability

To enhance the trustworthiness of the study, we applied Guba and Lincoln’s criteria, including credibility, dependability, confirmability, and transferability [24]. Credibility was enhanced through triangulation of data sources (i.e., senior, mid-level, and operational managers), member checking (participants reviewed and validated findings), prolonged engagement, iterative questioning during interviews, and peer debriefing. Confirmability was addressed through team-based coding, researcher reflexivity, and maintaining an audit trail of analytical decisions. Dependability was ensured by recording the coding process and analytic steps in MAXQDA.Transferability was strengthened by reporting participants’ demographic profiles and providing thick descriptions of the study setting and context. These strategies aimed to enhance the accuracy, transparency, and applicability of the study’s findings in both local and broader healthcare contexts.

## Results

The demographic characteristics of the study participants are presented in Table 1. The majority of participants were male [22], held a master’s degree or a professional doctorate [20], and had 10 to 15 years of work experience [18]. From the conducted interviews, a total of 375 initial codes were identified, which, after multiple reviews, refinements, and summarization based on similarity and relevance, were categorized accordingly. Tables 2 and 3 outline the challenges and crisis management strategies across the three domains of prevention, preparedness, and response.

**Table 1:** Demographic characteristics of interviewees

Variable	Frequency (%)
Gender	
Male	22(88)
Female	3(12)
Education Level	
Bachelor’s Degree	5(6)
Master’s Degree	11(44)
Professional/Doctoral Degree	9(36)
Work Experience	
More than 15 years	4(16)
10–15 years	18(72)
0–9 years	3(12)
Position	
Senior Health Officials (P1–P5)	5(20)
Assistant Health Directors (P6–P9)	3(12)
Department Managers (P10–P22)	12(48)
Service Center Directors (P23–P25)	3(12)

**Table 2:** Challenges in different stages of COVID-19 crisis management

Category	Subcategories	Code
Prevention	Awareness and Understanding of the Disease	Insufficient understanding of the disease’s risks
		Lack of awareness of disease characteristics
Preparedness	Planning	Weak risk assessment
		Delays in public notification and warnings
Response	Cultural-Social	Traditions and customs
		Public perception of the disease
		Disregard for health regulations and recommendations
	Political-Economic	Lack of trust in media and executive authorities
		Public’s economic situation
	Governance	Organizational structure
		Decision-making
		Constant changes in guidelines
	Intersectoral Cooperation	Lack of cooperation from other entities
		Unclear roles of other organizations during epidemics
		Weak intersectoral coordination

**Table 3:** Proposed strategies in different stages of COVID-19 crisis management

Category	Subcategories	Code
Prevention	Strengthening health and prevention	Strengthening the structure of the health sector
		Health education
Preparedness	Managing human and informational resources	Increasing the quality of human resources training
		Strengthening informational infrastructure
	Organizing and planning	Organizing equipment and personnel for crises
		Establishing a crisis and disaster unit
		Developing strategic and operational plans
Response	Coordination and decision-making	Increasing coordination and collaboration
		Improving decision-making in crises
	Managing human resources	Providing sufficient motivation
		Structuring and deploying human resources effectively during crises

## Challenges

### Prevention

#### Awareness and understanding of the disease

##### Insufficient understanding of the disease’s risks

The most significant challenge observed during the prevention stage was the insufficient understanding of the COVID-19 pandemic’s risks by officials. The negligence of the authorities toward the epidemic risk led to the failure to implement necessary actions to prevent its spread. The failure to quarantine infected provinces and cities at the beginning of the pandemic, as well as the movement of travelers from infected areas to different cities, contributed to the widespread transmission of the disease across the country.

“After the disease started in Qom and Tehran, people were scared, and most of the residents of those cities, or those who had gone for work, all returned to their hometowns. Our city was no different; since there aren’t job opportunities in our town, most people go to cities like Tehran for work, and everyone was coming back, and unfortunately, the cities were not quarantined, and travelers came in, worsening the situation” (P14).

On the other hand, the authorities’ indifference at the provincial and district levels, including the governorate and district administration, to the health sector’s warnings regarding necessary actions before the outbreak, such as communication and education, was another challenge for disease control expressed by interviewees.

“We wanted to start the necessary education regarding COVID-19 in early February. We even printed posters and banners, which are still available, but the governorate and district administration did not allow it. They told us to collect the educational materials. We went and explained the importance of education and prevention, but unfortunately, they said that it would create fear and tension among the people” (P15).

Interviewees also pointed out that despite the delivery of health protocols and the education on the importance of maintaining physical distancing, the indifference and negligence of election officials led to the further spread of the disease.

### Lack of awareness of disease characteristics

The characteristics of the disease, due to the nature of the virus, presented challenges in preventing the COVID-19 crisis. Interviewees noted that the constant changes in the symptoms and behavior of the disease reduced the ability to respond to it. The change in symptoms made it difficult to identify patients in time and implement quarantine measures for the infected, which contributed to the worsening of the pandemic. Additionally, the long incubation period of the disease, lasting up to 14 days, was another challenge in controlling the epidemic.

“However, considering that I had previously worked in the disease management unit over the past ten years, the world has not faced an emergency situation like this. At the health department level, before the disease entered Iran, the Ministry sent a national guideline, and we formed a committee and team based on that, specifying duties. But we had a different perspective on the disease and honestly didn’t anticipate how widespread the disease would become and how rapidly it would spread” (P12).

### Preparedness

#### Planning

##### Weak risk assessment

The main challenge that emerged during the preparedness phase for the COVID-19 epidemic was the weakness in planning for crisis management, which became apparent in two areas: weakness in risk assessment and delay in public communication and warning. The lack of epidemic forecasting, the low sensitivity of officials to its risk, and the lack of an accurate forecast about the future course of the disease led to insufficient preparedness to confront COVID-19.

“We didn’t have enough preparedness to face this disease. In fact, we were caught off guard because of the high volume of cases, and we didn’t have enough preparedness. Protective equipment and disinfectants weren’t procured in sufficient amounts, which caused problems later because we didn’t anticipate the disease with such intensity and spread. We thought it would be like other epidemics and infectious diseases that we had faced in previous years” (P7).

### Delays in public notification and warnings

The delay in public communication and warnings about the disease was another challenge raised by the interviewees. The lack of timely reporting of cases and the weak communication from the media and officials about the disease’s spread and transmission methods prevented adequate preparedness for responding to the crisis. Interviewees stated that the delay in declaring the cases in the country and even at the provincial level resulted in a lack of preparedness for responding to the disease. Also, the weak media coverage about the disease and its risks was a serious challenge for gaining the necessary preparedness.

“Unfortunately, due to political conditions or conflicting interests at the national level, the disease’s entry and the positive cases were declared late. They waited until the disease in Qom, for example, had already spread significantly, and a large number of people had contracted it. The same was true for Tehran, while an early public announcement should have been made. Universities should have been informed to prepare adequately. This disease has a 14-day incubation period. With the identification of a single suspicious case and public notification, many incidents could have been prevented, and at least some level of preparedness could have been achieved” (P2).

### Response

The challenges during the response phase consisted of five sub-categories and fourteen sub-elements. These sub-categories included cultural-social challenges, political-economic challenges, managerial challenges, and inter-organizational collaboration.

### Cultural-social challenges

#### Customs and traditions

Public gatherings organized by people in various forms were one of the obstacles to an effective response to the epidemic. Interviewees highlighted the severe family and tribal tensions, such as holding family reunions and visiting COVID-19 patients, which contributed to further transmission of the disease. Adherence to attending mourning ceremonies for those who died from COVID-19 and even wedding ceremonies were other cultural barriers to an appropriate response for controlling the epidemic.

“In our province, one of the problems was that most families were large, and due to tradition or respect, family connections were strong. Families frequently visited each other, even visiting COVID-19 patients, and they valued it highly. This led to gatherings and the transmission of the disease” (P2).

“A major issue was that attending mourning and burial ceremonies was very important for our people. The grieving family expected and hoped that all relatives would attend. People who didn’t attend these ceremonies were considered disrespectful” (P4).

#### Community attitude toward the disease

The community’s attitude toward the disease was another challenge in combating the COVID-19 crisis. Statements from interviewees revealed that fear of the disease and its stigma led to reluctance in getting tested for screening. The negative attitude toward infected individuals and fear of stigma led to the concealment of the disease by the infected person, which in turn caused further spread of the epidemic. The disbelief of some individuals in the existence of the disease posed another challenge in confronting the epidemic.

#### Disregard for health regulations and recommendations

The public’s disregard for health regulations and instructions was a major challenge to effectively confronting the COVID-19 epidemic. Non-compliance with health regulations and the lack of enforceable measures were further obstacles to responding to the COVID-19 epidemic. For example, shopkeepers and restaurant owners did not adhere to mandatory closure regulations:

“Our environmental health personnel would go to the shops and restaurants to seal them. The next day, the shop or restaurant owner would remove the seal by themselves, and no one would confront them. In some cases, when the environmental health personnel went for sealing, people started insulting and beating them” (P9).

### Political-economic factors

#### Weak trust in media and executives

Another challenge during the response phase was the weak public trust in media and executive agents. Contradictory messages from the media (including TV) about COVID-19 damaged the public’s trust in reports and advice from the media:

“People were seeing daily deaths around them due to COVID-19, and then officials came on TV and said, ‘We have five deaths in the province.’ Meanwhile, in our district alone, we had five deaths due to COVID-19. These contradictions made the public lose trust in the media, their statistics, information, and even their educational advice, making our job much harder” (P11).

#### Economic conditions of the people

Economic problems among the public were another obstacle in responding to the epidemic. Since many individuals rely on daily wages, if they contracted the disease, they did not follow quarantine protocols and instead went to work, thus helping to spread the disease further:

“Some people working in the private sector were either threatened with dismissal or not paid for the 14 days they missed work. So, they would hide their illness and go to work, which helped the disease continue spreading” (P11).

Market traders’ resistance to closing shops and commercial centers was also an issue. Early on in the pandemic, during the beginning of the Persian New Year, many market vendors, motivated by economic reasons, were reluctant to close their shops.

### Governance

#### Organizational structure

Several participants pointed out that the organizational structure of health crisis response was fragmented and unclear. This lack of structure led to duplication of tasks, overlapping responsibilities, and ineffective use of resources.

*“Sometimes three different departments would try to handle the same issue*,* while another issue was neglected entirely. There was no clarity about who should do what.”* (P10).

#### Decision-making

A recurring challenge mentioned was the centralization of decision-making, where local health managers lacked the autonomy to act promptly. This top-down approach delayed urgent responses.

*“We constantly had to wait for approval from higher-ups*,* even when the situation demanded immediate action. That cost us valuable time.”* (P3).

#### Constant changes in guidelines

Interviewees also expressed frustration over frequent and sometimes contradictory changes in official COVID-19 protocols. These changes created confusion among health workers and the general public.

*“We received new guidelines almost daily*,* sometimes contradicting the previous ones. Staff didn’t know which version to follow anymore.”* (P13).

#### Lack of cooperation from other entities

Participants emphasized the lack of active participation from key organizations such as municipal authorities, law enforcement, and NGOs. Their minimal engagement put extra pressure on the healthcare sector.

*“Despite repeated invitations*,* many agencies refused to collaborate. They said it was not their responsibility.”* (P4).

### Intersectoral cooperation

#### Unclear roles of other organizations

It was also noted that during the pandemic, the roles and responsibilities of various organizations were poorly defined, causing delays in implementing essential measures.

*“In meetings*,* nobody knew exactly what their role was. The instructions were vague*,* and everyone hesitated to act.” (P15)*.

#### Weak intersectoral coordination

A lack of structured coordination mechanisms across sectors further hindered effective crisis management. Participants recommended establishing formal communication channels to avoid fragmentation.

*“Every department was working in its own silo. We needed a unified command center to coordinate activities and share information in real-time.”* (P19).

#### Solutions

The solutions proposed by the interviewees have been categorized into the areas of prevention, preparedness, and response.

### Prevention

#### Strengthening the structure of the health sector

Many participants highlighted that Iran’s health sector had long prioritized treatment over prevention. The COVID-19 crisis underscored the need to strengthen preventive health services, especially at the PHC level. This included better resource allocation and institutional backing for health promotion activities.

*“For years we focused only on treatment. COVID showed us prevention needs the same attention*,* if not more.” (P5)*.

#### Health education

Health education was frequently mentioned as a foundational pillar in preventing disease spread. Participants advocated for using culturally appropriate materials and engaging local leaders to increase effectiveness.

*“We had to tailor our messages. General slogans didn’t work. When the village elder spoke*,* people actually listened.” (P12)*.

In the area of prevention, attention to the health sector, including health education and strengthening the health system for greater resilience in facing various crises, is crucial. Given that in recent years, the health sector and infectious diseases had been overlooked due to the focus on the treatment sector, the current crisis has shifted attention to prevention and health.

#### Preparedness

In the preparedness phase, human and informational resource management, organization, and planning are among the proposed solutions for managing epidemic crises.

### Human and informational resource management

#### Increasing the quality of human resources training

Interviewees stressed the importance of crisis-specific training for health workers. Many staff were not adequately prepared for pandemic conditions, especially in triage, infection control, and communication.

*“Our people didn’t know how to respond at first. They were trained for regular duties*,* not for a crisis of this scale.” (P8)*.

#### Strengthening informational infrastructure

Participants noted that existing data systems were not equipped for real-time crisis tracking. Strengthening platforms like the SiB system was seen as critical.

*“The SiB system helped*,* but it wasn’t fast or flexible enough. We needed real-time dashboards and automated alerts.” (P6)*.

#### Organizing equipment and personnel

The lack of pre-identified equipment and staffing plans delayed effective response. Managers suggested pre-arranged supply chains and crisis rosters.

*“We were caught unprepared. We didn’t know where the ventilators were or who was on duty.” (P1)*.

#### Establishing a crisis and disaster unit

A recurring proposal was the formal creation of crisis units at the provincial and district levels, equipped with trained teams and standard operating procedures.

*“We should have a standing crisis unit—not one we build from scratch in an emergency.” (P14)*.

#### Response

In the response phase, coordination and decision-making through improving and increasing inter- and cross-sectoral collaborations, as well as better decision-making during crises, were proposed as strategies for an adequate response to a crisis.

#### Increasing coordination and collaboration

Enhanced inter-departmental and intersectoral coordination was cited as essential. Respondents proposed joint crisis drills and cross-agency liaisons.

*“Each sector worked on its own. We needed shared objectives and a unified command structure.” (P11)*.

#### Improving decision-making in crises

Timely and decentralized decision-making mechanisms were seen as necessary to speed up local response.

*“Delays in approvals were a major issue. Local units need more authority to act quickly.” (P3)*.

#### Providing sufficient motivation

Many healthcare workers experienced fatigue and demoralization. Participants suggested incentives, peer support, and recognition programs.

*“We felt forgotten. Just a small bonus or a thank-you message would have gone a long way.” (P17)*.

#### Proper organization

Interviewees emphasized the importance of clearly defined roles and responsibilities during the response phase. This included maintaining rosters, schedules, and reporting lines.

*“The chaos wasn’t due to a lack of effort—it was disorganization. Nobody knew who was leading what.” (P9)*.

## Discussion

The crisis caused by the COVID-19 pandemic opened various management dimensions for health systems. Managers at various levels of health systems have gained substantial experience in managing this crisis. The aim of the present qualitative study was to identify the challenges and obstacles in responding to the COVID-19 crisis at the primary healthcare level. The results of this study indicated that managers at various levels of primary healthcare faced numerous issues and challenges. This analysis explored various factors in prevention, preparedness, and response to the disease. The findings showed that in the prevention phase, the failure to quarantine infected cities and disregard for health warnings were observed. challenges in the preparedness stage appeared to be the most critical and recurrent across interviews. Delays in planning, insufficient risk assessment, and lack of intersectoral coordination were reported more frequently than issues in the prevention or response phases. This suggests that future crisis planning should prioritize building robust preparedness mechanisms, particularly within primary healthcare structures. In responding to the disease, cultural practices like holding ceremonies and family gatherings continued, and there was a negative attitude toward the disease and neglect of health regulations. Politically and economically, mistrust of the media and government, and economic problems in adhering to quarantine regulations, were among the other challenges.

### Cultural-social factors

Excessive social interactions and participation in various gatherings were cultural challenges affecting the control and response to the COVID-19 pandemic. Studies conducted in Iran also reported cultural factors, such as holding mourning and wedding ceremonies and participating in gatherings, as threats to responding to the COVID-19 pandemic [25, 26]. Research in the Middle East also showed that holding frequent religious gatherings was one of the challenges to controlling COVID-19 in the region [27]. However, in Saudi Arabia, controlling gatherings and religious ceremonies was a factor that helped in controlling the disease in the country [28]. A study in Nigeria found that cultural-social behaviors, such as religious ceremonies, festivals, greetings, and funerals, weakened the adherence to COVID-19 protocols [29]. One of the main factors contributing to the fifth wave of the epidemic in Iran was the holding of various gatherings and attendance at ceremonies [30]. As evidence shows, cultural-social factors, especially religious and social gatherings, have had a significant impact on controlling COVID-19 in Iran and other countries. Research indicates that managing these gatherings and promoting adherence to health protocols among the public, especially in communities with specific cultural norms, is a key factor in the success of epidemic control strategies. To effectively face health crises, community-centered approaches that consider cultural differences are essential.

It is worth noting that operational managers were more vocal about cultural challenges in the field, such as dealing with public resistance and misinformation, whereas mid-level and senior managers focused more on the design and communication of culturally appropriate policies. A negative societal attitude toward the disease and a disbelief in its existence were other challenges in responding to the COVID-19 crisis. Participants in the qualitative study by Bijani and colleagues (2021) in Iran also did not believe in the disease and failed to adhere to health protocols. Some participants in the aforementioned study also regarded contracting the disease as taboo and tried to hide their infection [31]. A cross-sectional study in Kurdistan showed that participants viewed contracting COVID-19 as a social stigma [32]. In a qualitative study (2023) in Iran, perceived threats were reported as factors affecting public participation in managing the COVID-19 crisis. The study stated that inadequate perception of the disease and its seriousness became a barrier [26]. While in cross-sectional studies conducted in Egypt and Pakistan, participants showed good awareness of the disease and a positive attitude toward protective measures, with social media being the largest source of information and awareness among the public [33, 34]. A study conducted in India (2020) also reported that most participants had a positive attitude toward COVID-19, considering it a serious disease [35]. A cross-sectional study in Indonesia (2020) found that knowledge did not significantly impact COVID-19 prevention behavior, but attitude played a significant role in prevention, with attitude being the most influential variable in COVID-19 prevention [36]. In a study conducted in Iran (2020), results indicated that public education about the disease and follow-up by responsible organizations positively affected the attitudes of the elderly toward the disease and preventive measures [37]. Therefore, increasing public knowledge about COVID-19 is essential to changing people’s attitudes toward prevention to ensure proper performance. Policymakers and managers must pay special attention to the role of the community and the importance of education, information dissemination, and their involvement in controlling crises. The dissemination of accurate information during an epidemic and involving the community is critically important. Key community players, such as media, education, NGOs, and relevant governmental bodies, all play a vital role.

Public disregard for health regulations and education was another challenge raised by interviewees. People were not following health guidelines properly and did not take health recommendations seriously. The results of a qualitative study in Iran (2022) showed that one of the challenges in controlling the COVID-19 pandemic was public non-compliance with health protocols [38]. Another qualitative study in Iran (2022) showed that failure to adhere to health guidelines was a barrier to disease prevention. This lack of adherence was reported as influenced by social, cultural, and economic factors that need to be addressed by policymakers [39]. While a cross-sectional study in Jordan showed that many people adhered to health guidelines, including quarantine, indicating the effectiveness of government policies in addressing potential barriers [40]. Such findings suggest that the health sector cannot overcome challenges on its own without the informed participation of the public [41]. Therefore, one of the most important factors contributing to successful crisis management is public participation. Through media and awareness campaigns, public participation can be significantly impacted.

### Political-economic factors

Weak trust in the media and government was one of the factors contributing to public disregard for health warnings and protocols. In Italy, results showed that multiple conflicting messages from various media sources and delays in disseminating information by policymakers led to public mistrust of authorities and poor crisis management of COVID-19 [42]. In a review study (2021) across 57 countries, the results showed that public mistrust in government actions during the COVID-19 pandemic was a significant challenge [43]. A study in Spain (2020) indicated that the COVID-19 crisis led to public mistrust in the government, and those who followed official information sources had more trust in the government [44]. In Indonesia, the failure to meet people’s expectations and needs through government policies led to public distrust in the government’s response to COVID-19 [45]. A study in Nigeria also revealed that public trust in government policies affected the government’s ability to control the disease, facilitating its spread [46]. Studies show that public distrust in the media and governments is a serious barrier to managing health crises, especially during the COVID-19 pandemic. This distrust not only negatively impacts adherence to health protocols but can also contribute to the spread of the disease and the inefficiency of government policies. Perspectives on political and economic barriers varied among managerial levels. Senior managers mainly highlighted structural issues such as policy delays and lack of economic planning at the national level. Mid-level managers were more concerned with inconsistencies in the implementation of financial support policies. Operational managers, however, focused on the immediate consequences of economic hardship, such as citizens’ inability to comply with quarantine due to income needs and job insecurity. These layered viewpoints helped reveal how broader policies translated into local-level challenges and shaped public response behaviors.

Additionally, challenges related to misinformation and the lack of public trust can be understood through the lens of the Crisis and Emergency Risk Communication (CERC) model [47]. This model emphasizes the importance of consistent, culturally appropriate, and timely communication during crises to build public trust and influence behavioral [47].To increase public trust and improve crisis management, governments and media should focus on transparency in communications and providing accurate, timely information to the public. Additionally, establishing effective communication channels and engaging directly with the community can foster a sense of participation. Addressing people’s needs and expectations through surveys and consultation sessions is also essential. Furthermore, educational programs on health protocols can help improve understanding and encourage compliance with the guidelines. By implementing these actions, public trust can be enhanced, and crisis management can be more effective.

In this study, the economic status and livelihoods of people were identified as factors influencing their presence in crowded places and non-compliance with quarantine regulations. The necessity for work during the pandemic and the failure to pay salaries in private companies led individuals to show up at work even if they were sick. A study in Kurdistan also found that economic problems and lack of financial support from the government led to improper implementation of the two-week shutdown policy [48]. A study in Bangladesh (2020) also indicated that economic challenges were a major issue in managing the crisis in the country [49, 50]. A study comparing the performance of various countries in addressing the COVID-19 crisis showed that infrastructure barriers, such as economic weakness and government failure to provide financial aid to businesses, led to inefficient and passive responses in these countries [51]. The results suggest that the economic status and livelihoods of individuals directly influence their behavior in the face of health crises. Non-compliance with quarantine and presence in crowded areas may stem from financial needs and lack of government support. Therefore, these factors must be considered when designing and implementing health policies. To improve people’s livelihoods and adherence to health protocols during a crisis, governments should provide financial support packages for vulnerable groups to meet their needs. Furthermore, facilitating remote work conditions can help reduce presence in crowded places. Organizing public awareness campaigns about the risks of neglecting hygiene and collaborating with NGOs to provide supportive services are other effective measures. Investing in economic infrastructure and providing financial support for businesses can improve livelihoods and prevent more severe issues in the future.

Lack of transparency in the government’s reporting of disease statistics, delayed information about the start of the pandemic, and contradictory statements from officials in the media led to insufficient preparedness and delayed responses to the pandemic. A qualitative study in Iran indicated that during the first year of the pandemic, communication faced challenges such as lack of transparency, conflicting statements from officials, conflicting interests, and multiple information sources [52]. In another comparative study, findings revealed that effective management of the COVID-19 crisis requires government transparency and the provision of accurate and timely information to the public during a crisis [53]. Results from a study conducted in Spain showed that good governance during the COVID-19 pandemic necessitates media honesty and government transparency towards the public, which, in this country, was weak during the pandemic [54]. Other studies conducted in different countries also found that transparency and public trust in the government are crucial factors in managing the crisis [55, 56]. The government’s lack of transparency in providing disease statistics and insufficient communication at the start of the pandemic had a significant negative impact on preparedness and response to the crisis. Conflicting statements from officials and the abundance of information sources caused confusion and reduced public trust. This issue was clearly observed in various countries, including Iran and Spain, and highlights the importance of good governance and information transparency in managing health crises. To improve health crisis management and increase transparency, the government must continuously release accurate and timely data on diseases. Establishing a unified and reliable information channel could prevent confusion. Additionally, training officials in communication and transparency is essential. Effective cooperation with the media and the establishment of independent bodies to oversee government performance also helps to strengthen public trust. These actions can ultimately enhance the community’s preparedness to face future crises.

### Governance challenges

Inadequate government support for the implementation of laws and regulations from the COVID-19 Task Force, including the lack of oversight on the supply and pricing of personal protective equipment (PPE) and laboratory supplies, was a significant barrier to controlling the COVID-19 pandemic. Other studies also identified the inefficiency of laws as a challenge in managing the COVID-19 crisis [57]. Results from a study in Iran (2020) revealed that delays in decisive governance were the greatest challenge in policymaking for combating COVID-19 [58]. A study from Iran (2020) also pointed out that inadequate government oversight of the distribution and hoarding of protective equipment by profiteers was one of the factors leading to a shortage of protective gear among healthcare workers [59]. In a case study from Oman (2021), the results showed that an effective response to pandemics requires cooperation among all governmental sectors, including the government itself. This collaboration necessitates the involvement of and resource capacity from various sectors, including the private sector, non-governmental organizations, and civil society [60]. Duarte et al. (2020) stated in their study that to respond appropriately to the COVID-19 crisis, countries must have a strong supervisory structure to ensure that clear and serious guidelines are in place for crisis response [61].

Inadequate government support for enforcing the laws and decisions of the COVID-19 Task Force, especially regarding the oversight of the supply and pricing of PPE and laboratory equipment, has been identified as one of the main obstacles to controlling the COVID-19 pandemic. Delays in decisive governance and insufficient oversight of protective equipment distribution are other significant challenges in crisis management. From a theoretical lens, these findings can be interpreted within the framework of the WHO Health System Building Blocks, particularly with regard to the governance components [62]. For instance, delays in implementing clear regulations and weak enforcement align with governance gaps, while lack of training and preparedness reflects challenges within the health workforce. The findings of studies show that effective responses to health crises require comprehensive collaboration between governmental and non-governmental sectors, as well as the establishment of a strong supervisory structure. To improve health crisis management and control pandemics, the government must strengthen its oversight over the distribution and pricing of protective equipment and implement stricter laws to prevent hoarding. Additionally, inter-sectoral cooperation between the government, private sector, and NGOs is essential to ensure more effective crisis responses. Developing and implementing clear and decisive guidelines can enhance public trust, and ultimately, providing training for healthcare workers and the community on the optimal use of protective equipment will strengthen crisis response capabilities.

### Inter-sectoral cooperation

Lack of coordination in the implementation of decisions and regulations to control COVID-19 at the macro level between the Ministry of Health and the government, as well as at the lower levels between provinces and various organizations, including governorates and health networks, has been another challenge in responding to this crisis. The discontinuation of cooperation between organizations after several months of the outbreak and the failure to comply with health guidelines by different organizations have further hindered the health sector’s ability to control the disease. A qualitative study conducted in Iran also revealed that insufficient participation of non-governmental organizations (NGOs) and the absence of a communication network and constructive interaction between government institutions and NGOs were challenges in responding to the COVID-19 crisis in the country [63]. In another qualitative study in Iran, results showed that the involvement of citizens and other organizations, both directly and indirectly involved in pandemic prevention, was an important factor in effective crisis management [64]. A case study conducted in Kurdistan City revealed that weak inter-organizational communication and operational activities between various organizations were barriers to effective COVID-19 management in the city [48]. Findings from a qualitative study in Mazandaran reported that strong intra- and inter-organizational coordination contributed to effective management of COVID-19 [65]. The fragmentation and lack of coordination described in this study further reinforce the relevance of the WHO framework, where *service delivery* and *leadership/governance* blocks emphasize multisectoral collaboration and integration of care across public institutions and stakeholders.

Therefore, the lack of coordination and the discontinuation of cooperation between various institutions in implementing health regulations, especially at both the macro and micro levels, have created serious challenges in controlling COVID-19. Operational managers highlighted daily-level coordination issues with local institutions and NGOs, while mid-level managers emphasized gaps in procedural clarity. Senior managers, on the other hand, pointed to broader policy inconsistencies and the lack of sustained governmental mandates as key barriers to effective intersectoral collaboration. Additionally, the absence of NGO participation and the weakness in inter-organizational communication have adversely affected crisis management. In contrast, strong cooperation and coordination can aid in more effective management of the disease. To improve COVID-19 crisis management, it is essential for the Ministry of Health and the government to cooperate more and implement health guidelines effectively and cohesively. Furthermore, focusing on creating strong communication networks between governmental institutions and NGOs will facilitate the active participation of citizens and local organizations. Holding joint meetings and specialized working groups can help strengthen inter-organizational communications and increase the necessary coordination for disease control.

### Lack of clear and comprehensive guidelines

The lack of clear and comprehensive disease guidelines was one of the key challenges identified by the interviewees. According to the findings, the problem of not designing and drafting specific guidelines related to disease symptoms and transmission methods not only caused confusion among healthcare workers but also led to the public’s distrust of health personnel and their recommendations. The large volume of educational materials sent by the Ministry of Health, contradictory guidelines regarding the disease, and frequent changes to information and protocols were major problems faced by healthcare workers in responding to the COVID-19 crisis. A qualitative study conducted in Iran (2022) showed that weaknesses in disease-related guidelines, including incomplete and impractical preventive guidelines, were among the challenges causing non-compliance with health protocols [39]. Other studies also pointed out that the lack of sufficient information regarding the disease and the absence of a comprehensive health guideline were significant weaknesses in responding to the COVID-19 crisis [57]. A qualitative study from Iran (2021) revealed that the absence of specific guidelines during the COVID-19 pandemic led to feelings of uncertainty and confusion among operating room staff [66]. In another qualitative study from Iran (2020), the lack of a comprehensive and unified guideline to reduce or halt social contacts and urban mobility restrictions was mentioned as one of the challenges in combating COVID-19 [67].

The absence of clear and comprehensive disease guidelines, particularly concerning symptoms and transmission methods, has been one of the fundamental challenges in managing the COVID-19 crisis. This lack has not only caused confusion among healthcare workers but also increased the public’s distrust of health recommendations. The contradictory educational content and frequent changes to information and protocols are additional issues that healthcare workers have faced during the crisis. Findings suggest that weaknesses in guidelines and inadequate disease-related information have undermined adherence to health protocols.

To improve responses to health crises, the Ministry of Health must develop a comprehensive and clear guideline on the disease, including symptoms, transmission methods, and preventive protocols. Furthermore, coordination in disseminating information and reducing contradictions in educational materials can help increase public trust in health personnel. Regular training sessions for healthcare workers to update their knowledge and familiarize them with new protocols are also crucial. Additionally, creating mechanisms to receive community feedback on guidelines and protocols can improve the quality of healthcare services and their adherence.

### Limitations of the study

While this study provides valuable insights into the challenges and strategies for managing the COVID-19 crisis within the primary healthcare system of Lorestan, certain limitations should be acknowledged. One key limitation is the limited generalizability of the findings. As a qualitative study, the results are based on participants’ experiences and perspectives, which may not fully apply to other regions or healthcare systems with different organizational structures, policies, and socio-cultural contexts. The study provides in-depth insights, but its applicability to broader settings remains constrained. Another potential limitation is selection bias, stemming from the use of purposive and snowball sampling. While these methods were effective in identifying knowledgeable participants, they may have led to the inclusion of individuals with similar backgrounds and viewpoints, possibly overlooking more diverse perspectives. However, efforts were made to include a range of managerial levels to capture a comprehensive understanding of the issue. Additionally, one limitation of this study is its exclusive reliance on a qualitative approach. While phenomenology was appropriate for exploring the lived experiences and perspectives of PHC managers during the COVID-19 crisis, it limits the generalizability of findings to broader populations. Future research could employ a mixed-methods approach, combining qualitative insights with quantitative surveys, to enhance both depth and breadth of understanding. Researcher involvement in data collection presents a possible source of bias. Since one of the researchers conducted the interviews and was familiar with the study setting, there was a risk that their prior knowledge and experiences could have influenced data collection and interpretation. To mitigate this, multiple researchers were involved in the coding process, and validation of findings was sought through participant feedback and external expert review. Despite these limitations, the study contributes valuable knowledge to the field of primary healthcare crisis management. By identifying key challenges and proposing strategies for improvement, it provides a strong foundation for further research and policy development in similar healthcare settings.

### Policy and practice implications

Building on these findings, several practical recommendations can guide future interventions. At the policy level, governments should invest in institutionalizing preparedness protocols within PHC systems, ensure intersectoral coordination through shared operational frameworks, and allocate dedicated funding for emergency planning. At the practice level, continuous capacity-building for frontline workers, culturally adapted public education campaigns, and mechanisms for rapid information sharing are essential. These strategies, derived from the empirical insights of this study, are critical to enhancing the effectiveness and equity of health crisis responses in similar contexts.

## Conclusion

To enhance future crisis management, it is crucial for the Ministry of Health to develop clear and adaptable guidelines, improve coordination among stakeholders, and ensure consistent public communication. Strengthening health education, organizing preparedness training, and integrating technology for data management can also support better responses to future crises. Specifically, to increase public trust, authorities should prioritize transparent and timely communication, actively engage the community in planning and decision-making, and build feedback mechanisms to address public concerns. To strengthen inter-sectoral cooperation, it is essential to establish formal coordination frameworks, define clear roles and responsibilities across sectors, and implement joint simulation exercises to ensure operational readiness. Additionally, providing targeted economic support for vulnerable populations may help improve compliance with health protocols and public confidence in health policies. While no single approach can fully mitigate crisis challenges, a combination of these strategies can contribute to a more effective and resilient healthcare response system.

## Supplementary Information

Supplementary Material 1.

## Data Availability

The datasets generated and/or analyzed during the current study consist of qualitative interview transcripts containing potentially identifiable information from healthcare managers. Due to the sensitive nature of the data and ethical considerations related to participant confidentiality, the full transcripts are not publicly available. However, anonymized excerpts or additional information supporting the findings may be made available by the corresponding author upon reasonable request and subject to approval by the relevant ethics committee.
