# Supplementary material for: Crisis management of COVID-19 at the primary healthcare level: challenges and solutions – a qualitative study
Source: BMC Prim Care. 2026 May 21;27:267. doi: 10.1186/s12875-026-03379-2 (PMC13366947; doi:10.1186/s12875-026-03379-2)
Supplement: Supplementary file 1 — Supplementary Material 1. [file 12875_2026_3379_MOESM1_ESM.pdf]

Interview Guide

Study Overview

This study aims to investigate the challenges and solutions in crisis management at the primary healthcare (PHC) level during the COVID-19 pandemic, focusing on four key phases: **Prevention, Preparedness, Response, and Resilience.** The research is conducted as part of a dissertation at **Tehran University of Medical Sciences.** You have been selected as a participant in this study. Please review the following information regarding your involvement:

1. **Voluntary Participation:** Your participation is entirely voluntary, and you are free to withdraw at any point without any consequences.
2. **Ethical Considerations:** The researcher is committed to upholding ethical standards, and you may decline to answer any question if you wish.
3. **Interview Details:** The interview will last approximately **60 minutes** and may be conducted in person, online, or through written responses. The researcher may take notes, and with your consent, your responses will be audio-recorded.
4. **Confidentiality:** Your identity will remain confidential, and no personal information will be disclosed in any reports or publications. Any data used for future research will be anonymized.
5. **Use of Responses:** Some of your statements may be quoted directly or indirectly, but pseudonyms or codes will be used to ensure anonymity.
6. **Access to Findings:** If requested, the research team will provide a summary of the study's key findings after its completion.
7. **Ethical Responsibility:** Signing the consent form does not exempt researchers from their ethical obligations. The research team is committed to ensuring high-quality methods and responsible decision-making.
8. **Research Team:** All researchers share equal responsibility, and **Zahra Asadi Piri** serves as a representative of the research team.
9. **Ethical Approval:** This research has been approved by the **Ethics Committee of Tehran University of Medical Sciences** under the ethical approval code **IR.TUMS.SPH.REC.1399.138.**

- **Name of Interviewee:**
- **Organizational Position:**
- **Interviewee's Organization:**
- **Date and Time of Interview:**
- **Interview Location:**

Interview Questions

Section 1: Prevention (Minimizing Risks Before the Crisis)

1. Prior to COVID-19, what preventive strategies were in place at your healthcare facility for managing infectious disease outbreaks?
2. Were there any surveillance systems or early warning mechanisms to detect potential outbreaks?
3. How did your facility promote infection control measures such as hygiene practices and vaccination campaigns before the pandemic?
4. What role did public health education and community engagement play in preventing COVID-19 transmission?
5. What were the primary obstacles in implementing preventive measures at the PHC level?

Section 2: Preparedness (Readiness Before the Crisis Escalates)

6. Did your facility have a pre-existing emergency response plan for pandemics?
7. What training and resources were available to healthcare workers for pandemic preparedness?
8. How well-equipped was your facility in terms of medical supplies, personal protective equipment (PPE), and infrastructure before the outbreak?
9. Were digital health tools (e.g., telemedicine, electronic health records) in place to support pandemic preparedness?
10. What were the most significant gaps in your facility's preparedness for COVID-19?

Section 3: Response (Actions Taken During the Crisis)

A. Patient Management and Healthcare Delivery

11. What were the main challenges in diagnosing and treating COVID-19 patients at the PHC level?
12. How did your facility balance COVID-19 care with routine healthcare services?
13. How effective were the triage and referral systems for severe cases?
14. What strategies were implemented to ensure continued care for non-COVID patients?

B. Workforce and Operations

15. How did the COVID-19 crisis impact staff workload, well-being, and burnout?
16. Did your facility experience staff shortages, and if so, how were they managed?
17. What role did teamwork and collaboration between departments play in handling the crisis?
18. How did healthcare workers adapt to evolving protocols and new information during the pandemic?

C. Communication and Public Engagement

19. How did your facility communicate COVID-19 guidelines to healthcare staff and the public?

20. What were the biggest challenges in combating misinformation and maintaining public trust?
21. How effective was coordination with local and national health authorities in implementing COVID-19 policies?

D. Resource Allocation and Financial Support

22. How did your facility manage shortages in PPE, testing kits, and other critical supplies?
23. Was financial support from the government or external organizations adequate? If not, what additional resources were needed?
24. How did your facility adapt or innovate in response to resource limitations?

Section 4: Resilience (Long-Term Recovery and Future Preparedness)

25. What key lessons did your facility learn from managing the COVID-19 crisis?
26. What infection prevention and control measures have been strengthened post-pandemic?
27. What long-term strategies have been introduced to improve crisis preparedness at the PHC level?
28. How can PHC facilities enhance resilience against future pandemics or public health emergencies?
29. What policy recommendations would you suggest to improve crisis management in PHC settings?
30. What additional support (e.g., financial aid, training, technology) would help PHC facilities become more resilient in future crises?
